# Bromide of Potassium

**Published:** 1870-11

**Authors:** 


### Bromide of Potassium.

Dr. Wm. R. Whitehead, of New York, says: "There is no remedy which deservedly enjoys a higher repute than the bromide of potassium. In sthenic forms of headache, and in a number of conditions intimately associated with uterine complaints, this substance is invaluable, and an extended experience of it in these circumstances has thoroughly established my faith in its efficacy. But I am convinced that the bromide of potassium, like many other valuable remedies, is occasionally incautiously used. The indications for its administration would at first sight appear very plain, should the physiological dogma concerning it invariably obtain, namely, that the bromide of potassium diminishes cerebral

---

*A synopsis of this paper will be published in our next number.
† A thorough *disbeliever*, and if vigorously applied, *barbarous*. [Ed. Buffalo Med. Journal.

congestion. It is necessary to add, I think, that this remedy also produces cerebral congestion. The enormous doses which are sometimes given should excite the fear that an opposite effect from that intended might be induced, and that rapidly. The bromide of potassium should be given only in moderate doses to obtain the full therapeutic effect for the relief of cerebral congestion ; that the primary action of this substance is to diminish the amount of blood circulating within the cranium, but that subsequently, and particularly in very large or toxic doses, the opposite obtains ; in other words, that intracranial congestion is induced. Indeed I am inclined to believe that the *sedative* effects of the medicine are always attended with more or less cerebral congestion."—*American Journal Medical Sciences.*

